# Attributional Styles and Their Impact on Depressive and Anxious Symptoms in Italian Children: Insights from the Italian Children’s Attributional Style Questionnaire-Revised (CASQ-R)

**DOI:** 10.3390/pediatric16040096

**Published:** 2024-12-10

**Authors:** Simona Scaini, Silvia Grazioli, Ludovica Giani, Barbara Forresi, Stefano De Francesco, Marcella Caputi

**Affiliations:** 1 Sigmund Freud University of Milan, Via Ripa di Porta Ticinese 77, 20143 Milan, Italy; s.grazioli@milano-sfu.it (S.G.); giani.phd@milano-sfu.it (L.G.); b.forresi@milano-sfu.it (B.F.); defrancesco.phd@milano-sfu.it (S.D.F.); 2 Child and Adolescent Unit, Italian Psychotherapy Clinics, Corso San Gottardo 5, 20143 Milan, Italy; 3 Department of Life Sciences, University of Trieste, Via E. Weiss, 2-34128 Trieste, Italy; marcella.caputi@units.it

**Keywords:** attributional styles, psychopathological symptoms, children and early adolescents, Children’s Attributional Style Questionnaire-Revised, latent class analysis

## Abstract

Background: There is evidence that the tendency to adopt a peculiar pattern of causal inference, known as attributional style, is likely related to specific patterns of psychopathology among youth. Objective: This study aims to assess preliminary psychometric properties of the Italian Children’s Attributional Style Questionnaire-Revised (CASQ-R) and to explore the presence of any subgroups of children and early adolescents from the general population who might exhibit internally homogeneous and externally heterogeneous attributional styles through latent class analysis, delving into the potential sociodemographic, namely age and gender, and clinical differences among the identified classes of attributional styles. Method: A sample of 337 children (11.29 ± 1.76 years old, 169 females, and 168 males) was recruited and their attributional styles and depressive and anxious symptoms were analyzed. Results: Two distinct classes were defined using the CASQ-R items in a latent class analysis (LCA). In particular, high levels of depressive (Kruskal–Wallis chi-squared = 9.37, df = 1, Bonferroni-adjusted *p* = 0.002) and school phobia (Kruskal–Wallis chi-squared = 7.17 df = 1, Bonferroni-adjusted *p* = 0.037) symptoms were reported by children showing an internal, global and stable attributional style for negative events and an external, specific and unstable attributional style for positive events. Conversely, low levels of depressive and school phobia symptoms were reported by children showing the opposite attributional style. Conclusions: The identified classes shed light on distinct patterns associated with depressive and anxious symptoms, offering potential insights for targeted interventions.

## 1. Introduction

The individual propensity to adopt a particular pattern to interpret the causes behind an event has been referred to as one’s attributional style or explanatory style [1]. Attributional style is defined by three dimensions: internal–external, stable–unstable, and global–specific [2]. The internal–external dimension refers to whether the explanation for an event is grounded in the physical, behavioural, or cognitive characteristics of the self (i.e., internal) or is attributed to factors beyond an individual’s control (i.e., external). The stable–unstable dimension involves whether the cause for an event is seen as having lasting (i.e., stable) or temporary effects (i.e., unstable). Lastly, the global–specific dimension differentiates between causes that are perceived to have widespread, general impacts on the individual’s life (i.e., global) versus those that affect only specific areas of their life (i.e., specific).

Considerable evidence underscores the importance of attributional style in human functioning and well-being. Research conducted in adult populations demonstrated a significant association between attributional style and health outcomes [3], occupational outcomes [4], athletic performance [5], and many other relevant measures of functioning [6]. Notably, these findings extend to adolescents. For instance, Dweck et al. (1995) and Dweck and Leggett (1988) [7,8] have persuasively argued that the attributions made by youths after academic and social task failures constitute a critical determinant of their subsequent responses to those tasks. That is, how youths explain or attribute these events serves as a predictive factor for their future explanations or attributions of similar occurrences. Along the same lines, research conducted on children identified a connection between attributional style and both social and academic performance [9,10,11,12,13]. Moreover, children who link their social failures to internal and stable factors, like a perceived lack of ability, tend to retreat from social interactions and experience lower peer acceptance [14].

In a psychopathological framework, internal, stable, and global attributions for the causes of negative events have demonstrated significant implications for depressive symptomatology in children and adolescents [15,16,17,18]. Moreover, existing literature suggests that children with anxiety disorders tend to exhibit higher negative attributions (internal, global, stable) for adverse events compared to normal controls (for a review, see Bell-Dolan & Wessler, 1994; Caffo et al., 2005; Schleider et al., 2014) [19,20,21]. A major risk factor for both anxiety and depression is the perception of having no control over life events. This is closely linked to a hopeless attributional style, which is characterized by feelings of uncontrollability, uncertainty, and unpredictability about future events.

If the association between attributional style and internalizing symptoms has been ascertained by several meta-analyses [22,23], the direction of this link has not yet been clarified. Most of the existing longitudinal studies considered attributional style as the predictor, but this does not exclude the possibility that internalizing symptoms predict a specific attributional style. In support of this claim, an experimental study conducted by [24] showed that depressive symptoms were able to predict negative attributional style among college students. More recently, another study demonstrated that, after controlling for depressive symptoms, positive attributions for good events were predicted by low levels of social anxiety in 337 Italian children [25].

Another possibility that has not been tested yet is that a bidirectional link exists between attributional style and internalizing symptoms.

Finally, attributional style may act as a mediator between specific individual features and internalizing symptoms. Support for this latter possibility comes from a recent study conducted by [26], who showed that a depressogenic attributional style significantly mediated the relationship between self-discrepancies and anxiety.

An ongoing debate is still active on whether gender might play a pivotal role in the definition of attributional styles. If on one hand there is evidence of gender differences in the way males and females tend to causally explain either positive or negative situations, on the other hand, controversial results exist on which gender is more associated with a specific attributional style. As stated by [27], gender differences were supported as males showed a more pessimistic attributional style on the Total-Stability and Total-Globality dimensions than females, but not on the Total-Internality dimension. In particular, male adolescents were more likely to attribute negative events to stable causes and positive events to unstable ones than their female counterparts, whereas females displayed a greater tendency to explain negative events in global rather than in specific terms and the causes of positive events in the opposite direction. Similarly, Berndt et al. [28] identified women as more prone to make global and positive attributions for successful outcomes, but global and negative attributions for failures. On the other hand, LaForge et al. [29] found that men were more likely to make internal attributions than females.

Regarding age, more subtle differences emerged. In particular, older adults reported more interactive attributions in relationship situations and more dispositional attributions in negative ones, whereas younger adults made more interactive attributions for ambiguous events [30].

Overall, beyond the nature and the direction of the link between attributional style and adaptive/maladaptive functioning, early identification of dysfunctional attributional style seems to be particularly important, as potentially lifelong consequences can be prevented or mitigated through timely interventions.

The main measure of attributional style in children is the Children’s Attributional Style Questionnaire (CASQ) [31], which aligns with the reformulation of the learned helplessness model of depression in children [32] and is composed of 48 items. Several studies have consistently demonstrated that children making more internal–stable–global attributions for negative events and more external–unstable–specific attributions for positive events, as measured by CASQ, tend to report higher levels of depressive symptoms compared to their peers with the reverse attributional style [33].

In recent years, a revised version of the CASQ has been developed, composed of 24 items. The fact that children have a limited attention span and that the CASQ is often used in larger test batteries has led to the development of a shortened version [31]. The 24 items of the revised CASQ (CASQ-R) [34] were selected based on psychometric analyses (i.e., positive corrected item-total correlations and internal consistency reliability) conducted on the answers of 449 elementary school children who completed the original CASQ [35,36]. The psychometric properties of the CASQ-R were assessed in a sample of 1086 children aged 9 to 12 years, with equal numbers of boys and girls, and a balanced representation of African American and Caucasian children. About half of these children (*n* = 475) completed the CASQ-R again six months later. The findings indicated that, while the CASQ-R was somewhat less reliable than the original CASQ, it exhibited moderate internal consistency reliability and fair test–retest reliability. However, it showed equivalent criterion-related validity for self-reported depressive symptoms. Another study [37] involving 621 adolescents (184 males and 437 females), aged 11–18 years, found an adequate model fit for a one-factor solution for negative event items and a two-factor solution for positive event items. The reliability estimates for these factors were low but acceptable for the negative events and below recommended guidelines for the positive events. Nonetheless, to date, the CASQ-R is the most widely used measure of youth attributions. Since the CASQ-R has never been studied in Italian children, this study aims to assess the preliminary psychometric properties of the Italian CASQ-R. Furthermore, to delve deeper into the investigation of attributional styles within the general population of Italian-speaking children, we conducted a latent class analysis using the CASQ-R items. In light of these premises, we expected to find high levels of depressive and anxious symptoms among children showing high scores on the internal–global–stable attributional items for negative events and low levels of depression and anxiety among youth with high scores on the external–specific–unstable attributional items for positive events.

This approach allowed us to explore the presence of any subgroups of children who might exhibit internally homogeneous and externally heterogeneous attributional styles. Finally, we tested the mean differences in the target sociodemographic and clinical variables across the identified classes.

## 2. Method

### 2.1. Participants

A sample of 368 children and early adolescents attending primary (*N* = 4) and middle (*N* = 4) schools located in three medium-to-big cities in Northern Italy, namely Vigevano (PV), Milan, and Canegrate (MI), was recruited for this research project through convenience sampling, taking advantage of previously established research collaborations. After receiving a complete description of the study, 89% of parents and children/early adolescents agreed to participate through signed informed consent, thus leading to an effective study sample of 337 subjects. This latter sample size was reached after having excluded children and early adolescents who were declared to be affected by severe neurological/psychiatric disorders or who did not fully complete the test battery.

### 2.2. Procedure

The current research used pre-existing data gathered through a project focused on exploring the connection between socio-cognitive understanding and depressive symptoms.

The Italian Association of Psychology Ethical code, issued in 2015 and updated in 2022, states in Article 11 on page 9 that “Psychological research projects must be evaluated and approved by a Local Ethics Committee, if established, with a commitment to a rapid establishment where it does not yet exist” [38]. The present research dates back to 2014 when this ethical code had not yet been issued. However, although Italian laws and University guidelines for this type of study mandated no institutional review board approval, the study was conducted according to the Declaration of Helsinki [39]. Written informed consent was signed by parents of participating children, whereas children gave oral consent.

Participants were enrolled in the study upon the selection of schools and acceptance of the study by their Principals to whom the research study design was presented alongside the rationale, the main objectives, the methodology, and procedures of data collection and analyses.

The participants filled in self-report questionnaires in class during school time in one 50 min collective session. These questionnaires were aimed at assessing their attributional styles, as well as anxious and depressive symptoms in school-aged children and preadolescents. Participation in the study was primarily driven by individual willingness to contribute to expanding knowledge in the area of investigation and to support the achievement of the research goals, without any form of compensation or reward.

### 2.3. Measures

*Attributional style*. The Children’s Attributional Style Questionnaire-Revised (CASQ-R, see Appendix A) [31] consists of 24 double-choice items that address both positive (12 items) and negative (12 items) outcomes. Among the 12 positive events, 2 items focus on the internal-external dimension, 7 items evaluate the stable–unstable dimension, and 3 items pertain to the global–specific dimension. Similarly, for the 12 negative events, 3 items address the internal–external dimension, 6 items assess the stable–unstable dimension, and 3 items pertain to the global–specific dimension. Positive, negative, and overall (positive composite minus negative composite) scores can be derived. A lower positive composite score, along with a higher negative composite score, and a lower overall composite score indicate a more depressive attributional style. The child’s endorsed causal attributions are scored in a manner akin to the approach employed in the original CASQ.

*Depressive symptoms.* Depressive symptoms were measured through the Child Depression Inventory (CDI) [40,41]. The CDI comprises 27 items, each containing three self-report statements rated on a severity scale from 0 to 2, where 2 indicates a severe manifestation of a depressive symptom, and 0 signifies the absence of that symptom. The child is directed to fill out the CDI based on their emotions over the past two weeks. The overall score ranges from 0 to 54. In the current study, the internal consistency of the CDI was found to be satisfactory (Cronbach’s α = 0.82).

*Anxiety symptoms*. Anxiety symptoms were assessed with the Screen for Child Anxiety Related Emotional Disorders (SCARED) instrument [42,43]. It is a 41-item screening instrument for childhood anxiety disorders based on the DSM. Children/adolescents were asked to rate the frequency with which they experience each symptom on a 3-point Likert scale (0 = ‘almost never’, 1 = ‘sometimes’, 2 = ‘often’). According to the original factorial structure of the SCARED questionnaire, the 41 items can be divided into five subscales [44], namely panic/somatic anxiety, generalized anxiety, separation anxiety, social anxiety/social phobia, and school phobia. The overall score ranges from 0 to 82 with higher scores indicating greater levels of anxiety symptoms. Internal consistency was good in the present research (Cronbach’s panic/somatic anxiety: Cronbach’s α = 0.75; generalized anxiety: α = 0.72; separation anxiety: α = 0.71; social phobia: α = 0.73; School Phobia: α = 0.46; SCARED total: α = 0.86).

### 2.4. Data Analyses

Statistical analyses were carried out using SPSS 27 [45], and R version 4.2.2 statistical software [46] with the additional “poLCA” package for LCA [47]. An alpha level of 0.05 (two-tailed) was adopted as the criterion for statistical significance.

#### 2.4.1. Descriptive Statistics

Firstly, the descriptive statistics and frequency analyses of gender, age, and class attendance were performed for all participants to delineate the main characteristics of the children involved in the study. Moreover, item difficulty was checked [48].

#### 2.4.2. Correlation Coefficients

Point-biserial bivariate correlations of CASQ-R items with anxiety and depression were calculated. Moreover, the significance of between gender group correlation coefficients was tested.

#### 2.4.3. Psychometric Properties of CASQ-R

Psychometric properties of the CASQ-R, namely internal consistency obtained through the Kuder–Richardson reliability coefficient (KR20) and criterion-related validity obtained through the comparison with CDI scores, were analyzed [31].

#### 2.4.4. Latent Class Analysis

After verifying the model assumptions, we conducted a LCA to determine if two homogeneous groups of subjects could be identified within each sample. Specifically, LCA was performed on the dichotomous CASQ-R items; the decision to use two classes was guided by a priori hypotheses derived from a review of the literature [49]. Indeed, when determining the optimal number of classes, model fit indices such as AIC, BIC, g-test, and chi-squared are typically considered. However, as illustrated in [50] and [51], there is substantial evidence that BIC is particularly effective at identifying the correct number of latent classes, especially when datasets comprise a mix of categorical and continuous variables, with a predominance of the latter. Furthermore, these indices generally decrease with an increasing number of latent classes, as observed by [50], indicating that datasets with numerous indicators tend to favour more complex models due to the consistent decline in information criteria. In light of this, as previously demonstrated by our research group [52], and considering [51] recommendations to integrate a theory-driven approach, we opted for a 2-class solution. This decision is grounded in literature-based assumptions that the child population can be categorized into two primary attributional styles, each associated with distinct psychopathological symptoms. The two classes were then labelled based on the authors’ consensus after examining the descriptive profile of each class.

#### 2.4.5. Class Characteristics

Non-parametric between-class comparisons were carried out, considering, respectively, age, depressive, and anxiety symptoms as dependent variables. Lastly, Pearson’s chi-squared test was performed considering cluster membership and gender, to test for any associations between the two variables.

## 3. Results

### 3.1. Descriptive Statistics

The sample is composed of 337 children (11.29 ± 1.76 years old, 169 females and 168 males) attending the third (N = 87) and fifth (N = 73) year of primary school, and the second year (N = 177) of middle school.

All the items of the CASQ-R present a coefficient of difficulty that falls within the acceptable range (0.2–0.8), except for the items 1 (0.17), 7 (0.13), 22 (0.08), and 24 (0.91). Hence, these items were not considered in the following analyses.

### 3.2. Point-Biserial Bivariate Correlations

A series of point-biserial bivariate correlations were run, separately according to gender, among the following CASQ-R items (except for those that had item difficulties that did not fall within the acceptable range between 0.2 and 0.8 [48]): total depressive symptoms, and subtypes of anxiety assessed with SCARED.

Findings of the male sample revealed that item 2 of the CASQ-R positively and significantly correlated with generalized anxiety (rbp = 0.18, *p* = 0.02), whereas item 4 correlated with school phobia (rbp = 0.17, *p* = 0.03), and item 21 with panic disorder (rbp = 0.16, *p* = 0.04) and social anxiety (rbp = 0.20, *p* = 0.01). Conversely, item 9 (rbp = −0.19, *p* = 0.02), 18 (rbp = −0.16, *p* = 0.04) and 19 (rbp = −0.18, *p* = 0.02) showed negative and significant correlations with depressive symptoms. Item 16 negatively and significantly correlated with separation anxiety (rbp = −0.19, *p* = 0.02), whereas item 19 showed the same pattern of correlations with panic disorder (rbp = −0.23, *p* = 0.004), separation anxiety (rbp = −0.19, *p* = 0.02), and social anxiety (rbp = −0.20, *p* = 0.009). Among females, more correlations emerged between the CASQ-R’s items and the psychopathological investigated dimensions. In particular, item 2 negatively correlated with separation anxiety (rbp = −0.21, *p* = 0.008) and item 4 positively and significantly correlated with social anxiety (rbp = 0.22, *p* = 0.005), which was also the case for item 10 (rbp = 0.16, *p* = 0.04), item 15 (rbp = 0.20, *p* = 0.01) and item 18 (rbp = 0.17, *p* = 0.03). In the same direction, item 6 (rbp = 0.18, *p* = 0.02), item 11 (rbp = 0.16, *p* = 0.04), item 15 (rbp = 0.26, *p* < 0.001), and item 17 (rbp = 0.24, *p* = 0.002) correlated with depressive symptoms. Item 5 negatively and significantly correlated with social anxiety (rbp = −0.20, *p* = 0.01). Item 9 negatively and significantly correlated either with depressive symptoms (rbp = −0.29, *p* < 0.001) or panic disorder (rbp = −0.21, *p* = 0.007), generalized anxiety (rbp = −0.24, *p* = 0.002) and school phobia (rbp = −0.20, *p* = 0.01). Statistically significant and positive correlations emerged also between item 12, item 15 and item 18 with school phobia (rbp = 0.18, *p* = 0.02), panic disorder (rbp = 0.16, *p* = 0.04) and generalized anxiety (rbp = 0.17, *p* = 0.03), respectively. Finally, item 19 showed negative and significant correlations with depression (rbp = −0.31, *p* < 0.001) and item 21 with separation anxiety (rbp = −0.17, *p* = 0.03). Gender group comparisons highlighted that point-biserial coefficients between item 9 (z = 0.95; *p* = 0.34) and item 19 (z = 1.24; *p* = 0.22) of CASQ-R and depressive symptoms did not differ significantly in the two groups. 

### 3.3. Psychometric Properties of CASQ-R

Psychometric properties were calculated on the CASQ-R scales, excluding items that showed difficulties that did not fall within the acceptable range (between 0.2 and 0.8).

Internal consistency of CASQ-R: The internal consistency of CASQ-R was calculated using the Kuder–Richardson reliability coefficient (KR20), which is the statistical equivalent of Cronbach’s Alpha in cases of dichotomous items (usually scored as 0 or 1) for the overall composite (α = 0.364), positive (α = 0.316), and negative (α = 0.351) composite scores, as well as for the single CASQ-R subscales, namely positive internality (α = 0.285), positive stability (α = 0.207), positive globality (α = 0.104), negative internality (α = 0.260), negative stability (α = 0.272), and negative globality (α = 0.078).

Criterion-related validity of CASQ-R: Pearson’s bivariate correlations run on the entire sample are in line with those emerged in the validation study of CASQ-R by Thompson et al. (1998) [31]. Notably, CASQ-R positive (r = −0.296, *p* < 0.001), negative (r = 0.211, *p* < 0.001), and overall (r = −0.371, *p* < 0.001) composite scores correlated significantly and in the predicted directions with the children’s score for CDI.

Moreover, the CASQ-R positive composite score correlated significantly and in the predicted directions with the children’s score for SCARED panic disorder (r = −0.131, *p* = 0.018) and SCARED social phobia (r = −0.198, *p* < 0.001). The CASQ-R negative composite score correlated significantly and in the predicted directions with the children’s score for SCARED panic disorder (r = 0.131, *p* = 0.019), SCARED generalized anxiety (r = 0.136, *p* = 0.014), SCARED social phobia (r = 0.124, *p* = 0.025), and school phobia (r = 0.168, *p* = 0.002). Likewise, the CASQ-R overall composite score correlated significantly and in the predicted directions with the children’s score for SCARED panic disorder (r = −0.212, *p* < 0.001), SCARED generalized anxiety (r = −0.176, *p* = 0.001), SCARED social phobia (r = −0.222, *p* < 0.001), and school phobia (r = −0.206, *p* < 0.001).

To sum up, lower positive (i.e., a more depressive attributional style for positive events) and overall (i.e., more internal–stable–global attributions for negative events and more external–unstable–specific attributions for positive events) composite scores were associated with higher levels of depressive and anxiety symptoms in children; whereas higher negative composite scores (i.e., a depressive attributional style for negative events) were related to heightened self-reported symptoms of depression and anxiety.

Criterion-related validity of CASQ-R was also confirmed separately by gender and age categories. The emerged correlational patterns were in line with the point-biserial correlations depicted above, whereas the criterion-related validity results for younger and older children were the same as those for the whole sample.

### 3.4. Latent Class Analysis

Two classes were identified through LCA; the first consisted of 28% of the whole sample, whereas the second consisted of 72% of the subjects. Subjects referring to class 1 obtained a mode value of 1, which indicates the tendency to choose the items’ option “b” in the following CASQ-R items: 2, 3, 4, 6, 10, 11, 12, 13, 15, 16, 17, 18, 19 and 23; on the contrary, they obtained a mode value of 0, reflecting the opposite option “a”, in the remaining items. Thus, this class (representing the minority of the sample subjects) is characterized by an internal, global, and stable attributional style for negative events and an external, specific, and unstable attributional style for positive events.

Conversely, subjects referring to class 2 obtained a mode value of 1 in the following CASQ-R items: 5, 8, 9, 13, 16, 17, 18, 19, 20, and 23; on the contrary, they obtained a mode value of 0 in the remaining items. Thus, this class (representing the majority of the sample subjects) is characterized by an internal, global, and stable attributional style for positive events and an external, specific, and unstable for negative events.

These class characteristics are depicted in Table 1.

### 3.5. Class Characteristics

Individuals with an internal, global, stable attributional style for negative events and with an external, specific and unstable attributional style for positive events were classified as having a depressive and school phobic attributional style, whereas individuals with an internal, global, stable attributional style for positive events and with an external, specific and unstable attributional style for negative events were categorized as having a non-depressive and school phobic attributional style.

Depressive symptoms significantly differ between the two groups (Kruskal–Wallis chi-squared = 9.37, df = 1, Bonferroni-adjusted *p*-value = 0.002); class 1 showed significantly higher values of CDI, with a median of 12 (compared to a median of 9 in class 2). School phobia was also significantly higher (Kruskal–Wallis chi-squared = 7.17 df = 1, Bonferroni-adjusted *p*-value = 0.037) in class 1, with a median value of SCARED school phobia of 2.16, compared to a median of 1.70 in class 2. Other clinical or socio-demographic characteristics did not significantly differ between the two classes.

## 4. Discussion

The present research aimed to evaluate the psychometric properties of the Italian CASQ-R and to explore the presence of subgroups of children and early adolescents of the general population who might exhibit internally homogeneous and externally heterogeneous attributional styles. Moreover, we aimed to investigate potential sociodemographic and clinical differences among the identified classes of attributional styles.

Overall, the internal consistency of the CASQ-R composite scores was very low in the present study and the criterion-related validity assessed through correlation with CDI and SCARED scores, reflecting depressive and anxiety symptoms, respectively. However, existing literature concerning both CASQ and CASQ-R (both original version and translations) also reported mediocre values [31,37,53,54,55,56,57]. Therefore, a latent class analysis for dichotomous variables (i.e., LCA) was carried out to typify subjects according to their responses to single CASQ-R items, rather than considering the composite scores. Our findings have highlighted the existence of two classes of individuals. The first is characterized by an internal, global, and stable attributional style for negative events and by an external, specific, and unstable attributional style for positive events; the second one is represented by an internal, global, and stable attributional style for positive events and an external, specific, and unstable attributional style for negative events. Coherently with the existing evidence, those falling in class 1 show also higher depressive and anxiety symptoms [49]. It is well established that internal, stable, and global attributions for negative events have important implications for youth’s mental health problems [58].

### 4.1. Characteristics of Attributional Styles Associated with Depression and Anxiety

Our results are not directly comparable with those of other research, as no previous study used latent class analysis to look at attributional styles. Nonetheless, the features of the distinct classes are in line with the large amount of literature that has accumulated on the attributional styles that typically characterize depressed subjects [22,23,59,60]. In particular, the findings of the present study perfectly fit and are in line with the results reported by the meta-analysis conducted by [49] who emphasized that individuals with a tendency towards perceiving causes of failures and adverse events as being dependent by own characteristics, as well as viewing it as occurring consistently, repeatedly, and as generalizable across multiple situations, exhibited higher depressive symptoms compared to those who perceived the negative event as caused by external factors, specific and with a one-time occurrence. The effect size of the relationship between attributional styles and depression ranged from small to medium, with variety mostly explained by publication bias or by the absence of homogeneity examination among studies [22,23,27].

It is also worth mentioning that the same directional relationship was found between attributional styles and anxiety symptoms [21]. There is evidence that anxiety and related arousal patterns arise when uncontrollability and uncertainty are faced [61]. Therefore, habitually viewing negative events as being due to stable/unchangeable and global/generalized, and thus uncontrollable, causes may act as one of the main hallmarked risk factors for the development of anxiety either in adults [19,62] or youth [21]. The study by [63] offered further support of the link between internal–stable–global attributional style and anxiety, as it was encountered among children with a diagnosis of anxiety disorder but not among typically developing children.

### 4.2. Socio-Demographic Characteristics of Attributional Styles

Moreover, in this study, neither age nor gender differences emerged for CASQ-R classes, coherently with the results obtained in previous studies conducted on subjects of a similar age [31]. This finding might be grounded on the fact that children’s attributions may develop into relatively stable styles only in late childhood and early adolescence when significant differences are expected to arise [36].

With regard to gender, Ref. [64] as well as [65] stated that females are more likely to report more negative attributions than males and that those differences that typically emerge during adolescence remain across one’s lifespan. In contrast to these findings, our study did not reveal any gender discrepancies among attributional styles.

Nonetheless, we believe that gender should still be investigated in future research, as it could be an important factor especially when considering attributions interacting with depressive symptoms and/or anxiety [66,67,68,69,70,71,72]. Similarly, age must be taken into account when the comparison is between very different age groups, since people become increasingly selective in engaging cognitive resources and they can develop expert social knowledge across time [73,74].

### 4.3. Clinical Implications

Overall, the strength of our study is that the interesting groupings of subjects that emerged could pave the way to guide interventions aimed at reducing depressive symptoms starting from the school context by acting on attributional styles in children and adolescents. Such interventions could build on Dweck’s work conducted within the framework of the cognitive theories of depression, which focuses on the cognitive restructuring of attributions [75]. More recently, children’s beliefs/mindsets about the stability of self-concepts such as intelligence and personality received growing attention. Therefore, promising interventions could also target children’s mindset, with potential beneficial effects on well-being [76,77]. Another strength is that our analysis of the psychometric properties of the CASQ-R provide mental health professionals with a new tool able to early detect attributional styles strongly associated with depressive symptomatology among youth.

### 4.4. Limits and Future Perspectives

Although our study offers valuable insights, it is essential to acknowledge certain limitations. First, the generalizability of our findings to a broader population may be constrained due to the regional focus on Northern Italy. Secondly, the cross-sectional design of our study limits our ability to establish causation, and future research employing longitudinal approaches could provide a more nuanced understanding of the phenomenon. Specifically, research should better address the independent and interactive relationships among attributional styles, life events, coping and depressive symptomatology [78,79]. Moreover, latent class analysis allows typifying subjects involved in the recruitment and could lead to different results if different individuals are considered. Therefore, those findings should be confirmed with further research. Finally, even though our preliminary analyses excluded relevant age differences among all the attributional styles’ scales and depressive symptoms, caution should be taken in the interpretation of the results as younger children might have lower emotional awareness as well as emotional cognition than older children. Notably, as stated by [80], children at the end of primary school are more prone to have a global self-worth concept, whereas adolescents tend to have both positive and negative self characterizations that usually co-exist but do not merge into a single common self concept.

In summary, our study investigated attributional styles and depressive symptoms in a sample of Italian children using the CASQ-R; our findings contribute to the understanding of attributional styles in the Italian context. The identified classes shed light on distinct patterns associated with depressive and anxiety symptoms, offering potential insights for targeted interventions. Future research should employ longitudinal designs, diverse measurement methods, and broader participant inclusion to strengthen causal inferences and enhance generalizability. Additionally, evaluating the effectiveness of interventions targeting attributional styles could further inform preventive strategies for depressive symptoms in children.

## Figures and Tables

**Table 1:** Attributional styles’ characteristics of items referring to Class 1 and Class 2.

Class 1: Depressive and School Phobic Attributional Style			
	Internal Attributional Style	Global Attributional Style	Stable Attributional Style
Positive events			
Item 13	-	X	-
Negative events			
Item 2	X	-	-
Item 3	X	-	-
Item 4	-	X	-
Item 6	-	-	X
Item 10	X	-	-
Item 11	-	X	-
Item 12	-	-	X
Item 15	-	-	X
**Class 2:** **Non-Depressive And School Phobic Attributional Style**			
	**Internal Attributional Style**	**Global Attributional Style**	**Stable Attributional Style**
Positive events			
Item 5	X	-	-
Item 8	X	-	-
Item 9	-	-	X
Item 13	-	X	-
Item 16	-	X	-
Item 17	-	-	X
Item 18	-	-	X
Item 19	-	-	X
Item 20	-	X	-
Item 23	X	-	-

## Data Availability

Dataset available on request from the authors.

## Appendix A. CASQ-R Items

1. You score a “10” on a test.
  - I am intelligent.
  - I have a particular aptitude for the subject of the test.

2. Some people you know say they don’t like you.
  - Sometimes these people are unpleasant toward me.
  - Sometimes I am unpleasant toward them.

3. A good friend says they hate you.
  - My friend was in a bad mood that day.
  - I was not pleasant to my friend that day.

4. Someone steals money from you.
  - That person is not honest.
  - Many people are not honest.

5. Your parents compliment you for something you did.
  - I am very good at doing certain things.
  - My parents like some of the things I do.

6. You break a window.
  - I am not careful enough.
  - Sometimes I am not careful enough.

7. You work on a project with a group of kids, but the project doesn’t go well.
  - I don’t work well with people in that particular group.
  - I never work well in groups.

8. You make a new friend.
  - I am a friendly person.
  - The people I meet are friendly.

9. You are currently getting along well with your family.
  - It is usually easy to get along with my family.
  - Sometimes it’s easy to get along with me when I’m with my family.

10. You get a bad grade in school.
  - I am not a good student.
  - Teachers make tests difficult.

11. You bump into a door so hard your nose starts bleeding.
  - I wasn’t watching where I was going.
  - Lately, I’ve been distracted.

12. Your room is messy.
  - I didn’t clean my room that day.
  - I usually don’t clean my room.

13. Your mom cooks your favorite dish.
  - There are few things my mom does to make me happy.
  - My mom usually likes to make me happy.

14. The team you are on loses a game.
  - Team members don’t help each other when they play together.
  - That day, team members didn’t help each other.

15. You didn’t do your household chores.
  - I was lazy that day.
  - I am often lazy.

16. You go to an amusement park and have a lot of fun.
  - Amusement parks usually excite me.
  - Many activities usually make me have fun.

17. You go to a friend’s party and have fun.
  - My friend usually throws fun parties.
  - My friend threw a fun party that day.

18. A substitute teacher who likes you as a student is in class.
  - I behaved well during class that day.
  - I almost always behave well during class.

19. You make your friends happy.
  - I am usually a fun person to be around.
  - Sometimes, I am a fun person to be around.

20. You complete a difficult puzzle.
  - I am good at puzzles.
  - I am good at many things.

21. You try out for a team but don’t make it.
  - I am not good at sports.
  - The other kids who tried out are very good at sports.

22. You fail a test.
  - All tests are difficult.
  - Only some tests are difficult.

23. You score a penalty kick in a soccer game.
  - I found the right angle to kick the ball.
  - The goalkeeper wasn’t very strong.

24. You get the best grade on a research project.
  - The other kids in my class didn’t put much effort into their projects.
  - I put a lot of effort into my project.
